# Japanese longitudinal biomarker study in progressive supranuclear palsy and corticobasal degeneration: Clinical features of the first registered patients and short-term follow-up analysis

**DOI:** 10.1016/j.prdoa.2024.100279

**Published:** 2024-10-26

**Authors:** Hiroshi Takigawa, Ritsuko Hanajima, Ikuko Aiba, Takayoshi Shimohata, Takahiko Tokuda, Mitsuya Morita, Osamu Onodera, Shigeo Murayama, Kazuko Hasegawa, Aya M. Tokumaru, Hisanori Kowa, Masato Kanazawa, Tameto Naoi, Kenji Nakashima, Takeshi Ikeuchi, JALPAC study group

**Affiliations:** a Division of Neurology, Department of Brain and Neurosciences, Faculty of Medicine, Tottori University, 36-1 Nishi-cho, Yonago 683-8504, Japan; b Department of Neurology, NHO Higashinagoya National Hospital, 5-101 Umemorizaka, Meito-ku, Nagoya 465, Japan; c Department of Neurology, Gifu University Graduate School of Medicine, 1-1 Yanagido, Gifu 501-1194, Japan; d Department of Molecular Pathobiology of Brain Diseases, Kyoto Prefectural University of Medicine, 465 Kajii-cho, Kamigyo-ku, Kyoto 602-0841, Japan; e Division of Neurology, Department of Internal Medicine, Jichi Medical University, 3311-1, Yakushiji, Shimotsuke, Tochigi 329-0498, Japan; f Department of Neurology, Clinical Neuroscience Branch, Brain Research Institute, Niigata University, 1-757 Asahimachi-dori, Chuoku, Niigata 951-8585, Japan; g Departments of Neurology and Neuropathology (Brain Bank for Aging Research), Tokyo Metropolitan Institute for Geriatrics and Gerontology, 35-2, Sakae-chou, Itabashi-ku, Tokyo 173-0015, Japan; h Department of Neurology, NHO Sagamihara National Hospital, 18-1 Sakuradai, Minami-ku, Sagamihara 252-0392, Japan; i Department of Diagnostic Radiology, Tokyo Metropolitan Geriatric Hospital and Institute of Gerontology, 35-2, Sakae-chou, Itabashi-ku, Tokyo 173-0015, Japan; j Department of Neurology, NHO Matsue Medical Center, 5-8-31 Agenogi, Matue 690-8556, Japan; k Department of Molecular Genetics, Brain Research Institute, Niigata University, 1-757 Asahimachi-dori, Chuoku, Niigata 951-8585, Japan

**Keywords:** Prospective cohort study, Tauopathy, Atypical parkinsonism, Natural history, Biomarker

## Abstract

**Introduction:**

Progressive supranuclear palsy (PSP) and corticobasal degeneration (CBD) manifest with variable clinical features. We initiated a multicenter prospective registry study—the Japanese Longitudinal Biomarker Study in PSP and CBD—in November 2014 at 45 Japanese institutions to collect clinical information and biological samples to elucidate the natural courses and diagnostic biomarkers of PSP/CBD.

**Methods:**

Initial symptoms, clinical features, and scores (Progressive Supranuclear Palsy Rating Scale [PSPRS], Barthel Index, Mini-Mental State Examination, and Frontal Assessment Battery) of patients clinically diagnosed with PSP/corticobasal syndrome (CBS) at the first registration were analyzed. PSPRS score progression in the initial 8 years and predictive factors were examined.

**Results:**

As of October 2022, first registration had been conducted for 349 patients—57 with probable/possible Richardson’s syndrome (RS), 133 with possible CBS, 41 with overlapping CBS and PSP criteria (RS/CBS group), 20 with PSP subtypes other than RS, and 98 who did not fulfill the PSP or CBS criteria. Among the RS, CBS, and RS/CBS groups, the RS group exhibited the best scores. Initial symptoms of personality change and asymmetric onset were correlated with the total PSPRS score. The average PSPRS score increment by the second registration (n = 116 patients) was 11.8 in all three groups, and progression was correlated with cognitive dysfunction. Seventy patients died during the study period. The 5-year survival rate from onset was approximately 90 %.

**Conclusion:**

There were fewer severe clinical features in the RS group than in the CBS group. Cognitive dysfunction may be important in predicting clinical severity and disease progression.

## Highlights

• Japanese study collects data samples from 349 patients across 45 sites since 2014.
• Examining PSP/CBS patients’ initial symptoms, revealing differences among subtypes.
• PSPRS score and mortality of CBS patients are severe among the subtypes.
• Cognitive dysfunction may be a predictive factor of disease severity and progression.

## 1 Introduction

Progressive supranuclear palsy (PSP) was initially defined as a neurodegenerative disorder characterized by postural instability, vertical supranuclear gaze palsy, neck and upper trunk rigidity, and cognitive impairment (Richardson’s syndrome [RS] type) [1]. Subsequently, many clinical subtypes of PSP with different clinical features have been reported [2], [3], [4], [5]. Corticobasal degeneration (CBD) is another neurodegenerative disease characterized by unilateral asymmetric extrapyramidal symptoms and focal cortical syndromes, namely, apraxia, the alien hand sign, and cortical sensory loss [6]. Recently, CBD is used as a name based on pathological diagnosis, while corticobasal syndrome (CBS) is used as a name based on clinical diagnosis. The clinical diagnosis of PSP and CBD is challenging, and useful biomarkers to aid in diagnosing and predicting prognosis are urgently needed. The identification of these biomarkers necessitates prospective registry studies for collecting biospecimens and information from patients. However, only few prospective cohort studies in Western countries have been reported [7], [8], [9].

We initiated a multicenter prospective registry study, named the Japanese Longitudinal Biomarker Study in PSP and CBD (JALPAC), in November 2014 to collect clinical information and biological samples and determine the clinical features of Japanese patients with PSP/CBD. This study aimed to elucidate the natural progression of PSP/CBD and identify reliable diagnostic biomarkers thereof. Herein, we outlined the design of the JALPAC and analyzed the clinical features of the first registered patients based on the data collected from November 2014 to October 2022.

## 2 Methods

### 2.1 Design of the JALPAC

The JALPAC involves 45 Japanese institutions (Table S1) with movement disorder specialists. Clinical information, biospecimens (serum, plasma, cerebrospinal fluid, genomic DNA, and lymphoblastoid cell samples), and brain magnetic resonance imaging (MRI) data are collected according to standardized protocols (Supplement).

The study registers patients with clinically suspected PSP or CBS.　The registration criteria for PSP are: meeting the National Institute for Neurological Disorders/Society for PSP (NINDS-SPSP) diagnostic criteria for RS [10] or previously reported diagnostic criteria for the other clinical PSP subtypes such as PSP-parkinsonism (PSP-P); PSP-pure akinesia with gait freezing (PSP-PAGF); and PSP with cerebellar ataxia (PSP-C) [2], [3], [4], [11] (Table S2). Patients who meet the mandatory inclusion criteria for PSP but do not meet any exclusion criteria are also registered. The registration criteria for patients with CBS follow the modified version of the Cambridge CBS diagnostic criteria [12] or Armstrong’s CBD diagnostic criteria [13] (Table S2).

The clinical datasheet for first registration is designed to obtain information regarding sex, age of onset, age at registration, duration of illness, initial symptoms, clinical course until first registration, clinical features at first registration, and clinical scores (Progressive Supranuclear Palsy Rating Scale [PSPRS] [14], Barthel Index [BI], Mini-Mental State Examination [MMSE], and Frontal Assessment Battery [FAB]) (Table S3). The clinical datasheet for follow-up (re-registration) is meant to obtain the PSPRS and BI scores. Biological samples and brain MRI data are also collected at each registration. Re-registrations have been carried out annually for registered patients. For patients who do not re-register, we collected data on their current medical status, ongoing medical care, and potential transfer to another hospital or patient death from investigators via e-mail 1 year after the last registration. We intend to follow up with registered patients for as long as possible to establish an informative database of patients with pathologically confirmed PSP/CBD.

A written informed consent form, which was approved by the institutional review boards of all centers and the Ethics Committee of Niigata University (G2019-0021), was signed by all study participants.

### 2.2 Central diagnosis

**Fig. 1:**
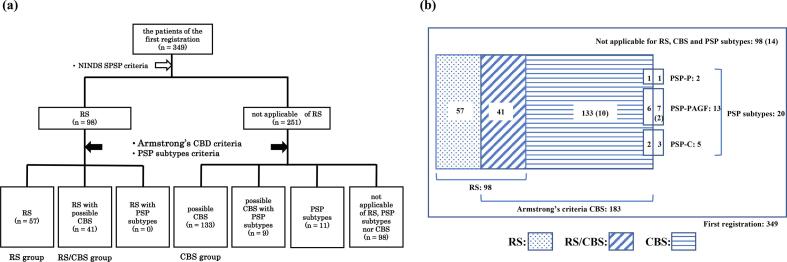
Classification of patients registered in the JALPAC. a) The flowchart for various PSP/CBS diagnosis and overlaps, b) The 349 patients are classified into three groups based on the central diagnosis—57 patients in the RS group; 41 who met both the RS and CBS clinical criteria in the RS/CBS group; and 133 who met the CBS clinical criteria, excluding those for the RS and PSP subtypes, in the CBS group. The numbers indicate the number of patients. The parentheses indicate the number of patients who met neither the RS criteria nor any RS exclusion criteria at the first registration but met the RS criteria at the final registration. JALPAC: Japanese Longitudinal Biomarker Study in Progressive Supranuclear Palsy and Corticobasal Degeneration; RS: Richardson’s syndrome; RS/CBS: Richardson’s syndrome/corticobasal syndrome; CBS: corticobasal syndrome.

All registered patients fulfilled the diagnostic criteria for RS, other PSP subtypes, or CBS (Table S2). Patients who met the NINDS-SPSP diagnostic criteria for probable/possible RS [10] were defined as having “clinical RS”, and patients who met the Armstrong CBD criteria for possible CBS were defined as having “clinical CBS”. Patients who met only the clinical RS criteria were categorized into “the RS group”; patients who met only the clinical CBS criteria but neither the clinical RS nor PSP subtype criteria into “the CBS group”; and patients who met both the clinical RS and CBS criteria into “the RS/CBS group”. We evaluated the patients in these three groups (Fig. 1).

### 2.3 Clinical features of the RS, RS/CBS, and CBS groups at the first registration

First, we analyzed the differences in clinical features at the first registration among the three groups. The annual change in PSPRS and BI scores from onset to the first registration was calculated as the score divided by disease duration. Differences in sex and initial symptoms were analyzed using the chi-square test. Differences in age of onset, age at registration, duration of illness, clinical scores, and annual changes in PSPRS and BI scores were analyzed using the Kruskal–Wallis and Mann–Whitney U tests. Bonferroni correction was used to adjust the probability as a post-hoc analysis.

The forced-entry and step-wise methods were used to select initial symptoms for inclusion as independent variables to identify the initial symptoms correlated with the total PSPRS score. The correlation between PSPRS and other scores (BI, MMSE, and FAB) was assessed using Pearson’s correlation coefficients. For all analyses, p < 0.05 was considered significant.

### 2.4 Clinical changes between the first registration and re-registrations

Changes in PSPRS scores between the first registration and re-registrations until October 2022 were analyzed using the Kruskal–Wallis test. Bonferroni correction was used to adjust the probability as a post-hoc analysis. Stepwise multiple regression analysis was used to identify clinical features at the first registration that predicted worsening PSPRS scores between the first and second registrations. The PSPRS score was set as the dependent variable, while the PSPRS, MMSE, and FAB scores were set as the independent variables at the time of the first registration.

Patients who not met RS criteria without any exclusion criteria at the first registration were reassessed at re-registration to determine if they met the NINDS-SPSP diagnostic criteria.

### 2.5 Analysis of deceased patients

Plots of overall survival after onset were generated using Kaplan–Meier analysis, and mortality rates were compared using the log-rank test. Data were censored at the period during which survival was confirmed. We also calculated the sensitivity and specificity of the clinical diagnoses of NINDS-SPSP criteria for PSP and Armstrong CBD criteria for CBD at the first registration. The threshold for statistical significance was set at p < 0.05.

## 3 Results

### 3.1 Diagnosis of registered patients

The first registration was conducted for 349 patients between November 2014 and October 2022. A total of 361 re-registration (follow-up) were conducted (the second registration: 166 patients; the third registration: 94 patients; the fourth registration: 50 patients; the fifth registration: 30 patients; the sixth registration: 13 patients; the seventh registration: 6 patients; the eighth registration: 2 patients) within the 8-year period. We obtained information over e-mail to confirm that the 73 patients who did not re-register 1 year after the first registration had transferred to another hospital or were lost to follow-up. No information was available for other patients. The rate of second registration was 47.6 % (166/349), and that of study outcome confirmation 1 year after the first registration was 68.5 % (239/349).

Concerning the central diagnosis at the first registration (Fig. 1), the clinical NINDS-SPSP diagnostic criteria for RS were fulfilled by 98 patients, of whom 50 met the NINDS-SPSP probable RS criteria, and 48 met the possible RS diagnostic criteria. Overall, 2 patients met the criteria for PSP-P; 13 for PSP-PAGF; and 5 for PSP-C. No patients met the Armstrong probable CBD criteria. Meanwhile, 183 patients met the Armstrong possible CBD criteria; of them, only three met the modified Cambridge CBS version criteria. There was an overlap of multiple diagnostic criteria in 50 patients (14.3 %)—41 patients met the clinical criteria for both RS and CBS (the RS/CBS group), and 9 patients met the CBS criteria and the criteria for another PSP subtype (except RS; PSP-P: 1 patient, PSP-PAGF: 6 patients, PSP-C: 2 patients). The remaining 98 patients did not meet any of these diagnostic criteria. Consequently, the RS and CBS groups involved 57 and 133 patients, respectively.

### 3.2 Clinical features at first registration

Sex, age of onset, age at registration, and duration of illness were not significantly different among the three groups at the time of the first registration. The PSPRS score was the highest in the CBS group (p < 0.01). There was no difference in the item of ocular motor between the RS group and the CBS group, but significant differences were found in the items of history, mentalization, and limb motor, which influenced the severity of PSPRS. However, the annual progression in PSPRS score from onset to the first registration did not differ significantly among the three groups. The BI scores at the first registration were also the lowest in the CBS group (p < 0.01). Moreover, the annual reduction rate of BI scores from onset to the first registration was the highest in this group (p < 0.05). Cognitive function scale scores were significantly worse in the CBS group than in the RS group (MMSE: p < 0.01; FAB: p < 0.01) (Table 1).

**Table 1:** Patient characteristics and initial symptoms at the first registration.

	RS group				RS/CBS group				CBS group				p-Value	Post-hoc (p < 0.05)
N (male/female)	57 (37/20)				41 (24/17)				133 (69/64)				0.24*	
Age at onset (years)	70.2 ± 7.2				66.8 ± 7.8				68.5 ± 7.4				0.09#	
Age at first registration (years)	74.1 ± 7.0				71.8 ± 7.5				73.2 ± 6.9				0.35#	
Disease duration (years)	3.9 ± 3.5				4.9 ± 3.6				4.7 ± 3.4				0.08#	
PSPRS score	30.5 ± 14.3				42.0 ± 20.0				43.6 ± 23.1				< 0.01#	RS/CBS vs. CBS, RS vs. CBS
History score	6.9 ± 4.1				9.1 ± 5.4				10.3 ± 6.1				< 0.01#	RS vs. CBS
Mentation score	2.4 ± 2.5				5.2 ± 3.8				5.4 ± 4.2				< 0.01#	RS/CBS vs. CBS, RS vs. CBS
Bulbar score	2.6 ± 2.1				3.3 ± 2.4				3.1 ± 2.5				0.38#	
Ocular motor score	5.3 ± 3.4				7.7 ± 4.0				6.5 ± 5.1				< 0.05#	RS vs. RS/CBS
Limb motor score	3.7 ± 2.2				5.9 ± 3.2				6.9 ± 3.9				< 0.01#	RS/CBS vs. CBS, RS vs. CBS
Gait and midline score	9.6 ± 4.8				10.8 ± 5.6				11.2 ± 6.3				0.27#	
BI score	67.7 ± 29.8				57.4 ± 37.3				50.5 ± 36.2				< 0.01#	RS/CBS vs. CBS, RS vs. CBS
Annual PSPRS progression rate from onset (per year)	11.2 ± 8.3				12.3 ± 7.6				12.7 ± 8.5				0.28#	
Annual BI reduction rate from onset (per year)	−9.9 ± 11.9				−9.7 ± 10.0				−13.2 ± 12.3				< 0.05#	RS/CBS vs. CBS, RS vs. CBS
MMSE score	24.5 ± 4.8				22.1 ± 6.2				20.3 ± 7.7				< 0.01#	RS vs. CBS
FAB score	12.0 ± 3.1				9.3 ± 4.6				9.0 ± 4.4				< 0.01#	RS vs. CBS
Initial symptoms	Present	Absent	NR	Present in cohort (%)	Present	Absent	NR	Present in cohort (%)	Present	Absent	NR	Present in cohort (%)		

Falls	33	23	0	58.9	22	19	0	53.6	61	70	2	46.5	0.28*	
Bradykinesia	28	27	2	50.9	24	16	1	60.0	69	62	2	52.7	0.51*	
Gait disturbance	39	17	1	69.6	34	6	1	85.0	79	54	0	59.4	< 0.01*	RS/CBS vs. CBS
Tremor	8	48	0	14.3	7	31	3	18.4	22	110	1	16.7	0.86*	
Ocular symptoms	13	41	3	24.1	16	23	2	41.0	25	103	5	19.5	< 0.05*	RS/CBS vs. CBS
Speech disturbance	19	38	0	33.3	24	17	0	58.5	55	76	2	42.0	< 0.05*	RS vs. RS/CBS
Dysphagia	11	46	0	19.3	7	34	0	17.1	25	106	2	19.1	0.95*	
Cognitive decline	13	43	1	23.2	22	18	1	55.0	48	84	1	36.4	< 0.01*	RS vs. RS/CBS
Hallucination	0	57	0	0.0	0	41	0	0.0	9	122	2	6.9	< 0.05*	RS vs. CBS
Abnormal behavior	0	57	0	0.0	4	37	0	9.8	11	120	2	8.4	0.07*	
Personality change	2	55	0	3.5	12	29	0	29.3	25	105	3	19.2	< 0.01*	RS vs. RS/CBS, RS vs. CBS
Depression	2	54	1	3.6	5	35	1	12.5	5	125	3	3.8	0.08*	
Apraxia	1	56	0	1.8	5	36	0	12.2	33	98	2	25.2	< 0.01*	RS vs. CBS
Traffic accident	6	50	1	10.7	5	34	2	12.8	19	108	6	15.0	0.73*	
Urinary disturbance	10	47	0	18.8	5	36	0	12.2	23	107	3	17.7	0.70*	
Dizziness	2	55	0	3.5	0	41	0	0.0	1	130	2	0.8	0.23*	
Asymmetric onset	13	40	4	24.5	17	21	3	44.7	69	55	9	55.6	< 0.01*	RS vs. CBS
Unilateral onset	10	43	4	18.9	11	27	3	28.9	58	66	9	46.8	< 0.01*	RS vs. CBS

Data are presented as the number or the mean ± standard deviation.

^#^Kruskal–Wallis test, post-hoc Bonferroni correction

*Chi-square test for analysis as well as post-hoc correction

NR: not recorded, PSPRS: Progressive Supranuclear Palsy Rating Scale, BI: Barthel Index, MMSE: Mini-Mental State Examination, FAB: Frontal Assessment Battery,

RS: Richardson’s syndrome, RS/CBS: Richardson’s syndrome/corticobasal syndrome, CBS: corticobasal syndrome.

Regarding the initial symptoms at the time of the first registration, the frequencies of hallucination (p < 0.05), apraxia (p < 0.01), asymmetric onset (p < 0.01), and unilateral onset (p < 0.01) were higher in the CBS group than in the RS group. The frequency of personality change (p < 0.01) was lowest in the RS group. Gait disturbance (p < 0.01) and ocular symptoms (p < 0.05) were more frequent in the RS/CBS group than in the CBS group. Meanwhile, speech disturbance (p < 0.05) and cognitive decline (p < 0.01) were more frequent in the RS/CBS group than in the RS groupTable 1.

The results of the multiple regression analysis showed that the PSPRS score at the first registration was correlated with age, RS/CBS subgroup, CBS, and the symptoms of personality change and asymmetric onset (R^2^ = 0.405, p < 0.0001) (Table 2). Additionally, the PSPRS score at the first registration was strongly correlated with the BI (R = 0.874), MMSE (R = 0.701), and FAB (R = 0.567) scores (Fig. S1).

**Table 2:** Factors associated with progression of PSPRS score: linear regression analysis results.

**PSPRS score at first registration^a^**			**Change in PSPRS score between the first and second registrations^b^**		
	**Regression coefficient**	**p-Value**		**Regression coefficient**	**p-Value**
Age	0.462	< 0.05	Age	−0.173	0.27
Sex	3.460	0.21	Sex	3.565	0.08
Group			Group		
RS	1	−	RS	1	−
RS/CBS	10.535	< 0.05	RS/CBS	−1.133	0.72
CBS	13.340	< 0.01	CBS	0.420	0.85
Personality change	10.100	< 0.01	Grasping/imitation/utilizing behavior	−2.231	< 0.05
Asymmetric onset	−8.107	< 0.01	Voluntary upward command movement in ocular motor	−2.028	< 0.05
			Voluntary downward command movement in ocular motor	2.903	< 0.01
			Sitting down	−2.401	< 0.01
			MMSE score	0.768	< 0.01
			FAB score	−1.157	< 0.01

Adjusted for age, sex, and clinical subtype.

a: Multiple regression analysis correlation coefficient = 0.405, p < 0.0001.

b: Multiple regression analysis correlation coefficient = 0.655, p < 0.0001.

PSPRS: Progressive Supranuclear Palsy Rating Scale, RS: Richardson’s syndrome, RS/CBS: Richardson’s syndrome/corticobasal syndrome, CBS: corticobasal syndrome.

### 3.3 Clinical changes from the first registration

**Fig. 2:**
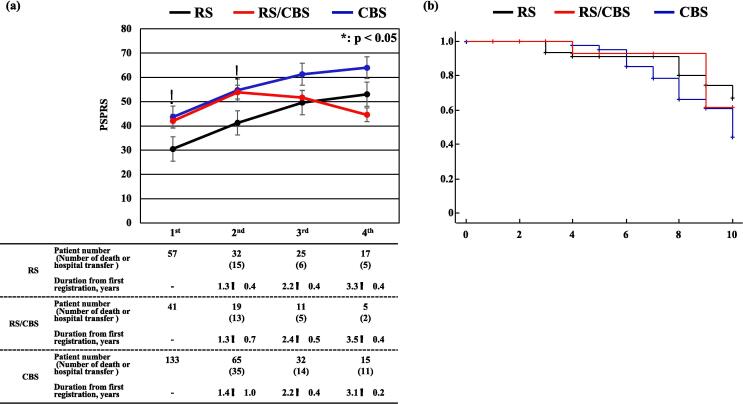
Results of clinical analysis after the first registration. a) The PSPRS scores in the RS, RS/CBS, and CBS groups at each registration are analyzed. The PSPRS scores until the second registration are higher in the RS/CBS and CBS groups than in the RS group. However, there are no significant differences in PSPRS scores among the three groups after the third registration. b) Kaplan–Meier plot illustrating the differences among the RS, RS/CBS, and CBS groups for 10 years after onset. Survival rates in the CBS group tend to be lower than those in the RS and RS/CBS groups. Y-axis: proportion of patients alive (surviving) at a given time. X-axis: survival in years. vertical dash: subject is censored. PSPRS: Progressive Supranuclear Palsy Rating Scale; RS: Richardson’s syndrome; RS/CBS: Richardson’s syndrome/corticobasal syndrome; CBS: corticobasal syndrome *: Kruskal–Wallis test and Bonferroni post-hoc correction, p-value < 0.05, RS vs. RS/CBS and CBS, bar: standard error.

The average increase in PSPRS score between the first and second registrations was 11.8 (12.5 in the RS, 10.7 in the RS/CBS, and 11.3 in the CBS groups). There was no significant difference in the increase in scores among the groups. At the second registration, the PSPRS score was the best (i.e., lowest) in the RS group. The number of patients followed-up after the third registration was smaller in the RS/CBS group than in the RS and CBS groups. This was possibly because the rates of death and transfer to another hospital tended to be higher in the RS/CBS group (RS group: 18.8 %; RS/CBS group: 26.3 %; CBS group: 21.5 %) (Fig. 2a, Table S4).

For the RS/CBS patients who dropped out between the second and third registrations, the average PSPRS score at the second registration was as high as 68.6. The progression in PSPRS score between the first and second registrations was positively correlated with voluntary downward command movement in ocular motor symptom and the FAB score at the first registration. Meanwhile, it was negatively correlated with grasping/imitation/utilizing behavior, voluntary upward command movement in ocular motor, and sitting down symptoms and MMSE score at the first registration (multiple regression analysis: R = 0.655, p < 0.01) (Table 2).

Twenty-six patients did not fulfill the RS criteria without any RS exclusion criteria at the first registration (CBS: 10 patients, PSP-PAGF: 2 patients, neither RS nor CBS: 14 patients). Among them, 10 patients were re-registered, and 4 of these 10 patients ([at the first registration] CBS: 1 patient, PSP-PAGF: 1 patient, neither RS nor CBS: 2 patients) met the criteria for RS at the final registration. Thus, the number of patients who fulfilled the RS criteria increased with disease progression.

### 3.4 Mortality rates and pathological diagnoses in autopsy cases

Kaplan–Meier and the log-rank analyses for 10 years from onset revealed that survival rates tended to be lower in the CBS group than in the RS and RS/CBS groups (Fig. 2b). The 5-year survival rate was approximately 90 % in all groups, which was better than the rate reported for PSP in Japan in 2005 [15]. The median survival time of CBS was 10.0 years. After the first registration, 70 patients died, and 21 of them underwent pathological autopsy. The pathological diagnosis confirmed PSP in 11 patients (clinical diagnoses: RS, 2 patients; RS/CBS, 2 patients; CBS, 6 patients; not applicable for RS/CBS, 1 patient) and CBD for 3 patients (clinical diagnoses: CBS, 1 patient; PSP-P and CBS overlap, 1 patient; not applicable for RS/CBS, 1 patient) (Table S5). Among the remaining seven patients, two patients were determined as having had Alzheimer’s disease (clinical diagnosis: RS/CBS, one patient; CBS, one patient) and one patient each as having had Lewy body disease (clinical diagnosis: CBS), frontotemporal lobar degeneration with TAR DNA-binding protein of 43 kDa proteinopathy (FTLD-TDP; clinical diagnosis: CBS), FTLD-TDP type B + Lewy body dementia, Kii-parkinsonism-dementia complex (clinical diagnosis: CBS), and FTLD (clinical diagnosis: not applicable for RS/CBS). The six patients clinically diagnosed with CBS and PSP pathology met the mandatory NINDS-SPSP diagnostic criteria but also met some exclusion criteria for RS (alien limb syndrome, cortical sensory deficits, and focal frontal/temporoparietal atrophy in five patients; severe, asymmetric parkinsonian signs in four patients; hallucinations or delusions unrelated to dopaminergic therapy in one patients; cortical dementia of Alzheimer’s type in one patients, and neuroradiologic evidence of relevant structural abnormality in one patients). The clinical diagnostic sensitivity and specificity were 36.4 % (4/11) and 90.0 % (9/10) for PSP and 66.7 % (2/3) and 22.2 % (4/18) for CBD, respectively (Table S5).

## 4 Discussion

The JALPAC is the first multicenter prospective study on possible PSP and CBD biomarkers in Japan, and more than 300 patients with possible PSP/CBD have been successfully enrolled in the first 8 years. The number of patients with clinically possible RS (n = 98) is larger than those in previous prospective cohort studies [16], [17]. We obtained data on the clinical courses of Japanese patients with possible PSP/CBS and compared their progression and mortality rates with those reported for Western patients [8], [9].

### 4.1 Clinical features of the RS, RS/CBS, and CBS groups at the time of first registration

Concerning the central diagnosis, the largest number of patients fulfilled the possible CBS criteria, and many patients met both the possible RS and possible CBS criteria. The patients who met overlapping criteria accounted for approximately 50 % of the patients with possible PSP and approximately 25 % of those with possible CBS. In our registry, a few CBS patients showed cortical sensory deficit (22.4 %; 41/183) or alien limb phenomena (9.8 %; 18/183). Therefore, no patient met the Armstrong probable CBS criteria, and only three patients met the probable CBS criteria in the modified Cambridge CBS version.

In addition, only two patients met the PSP-P criteria thus far. This number was smaller than that of a previous report [2] in which 33 (32 %) of the 103 patients pathologically diagnosed with PSP were diagnosed with PSP-P. However, clinical features of the PSP-P patients became similar to those of PSP-RS patients 2 years after disease onset [2]. Therefore, a proportion of PSP-P patients who registered more than 2 year after disease onset could be diagnosed with PSP-RS in JAPAC, and this could be one reason for the discrepancy of the number of PSP-P.

Among the RS, RS/CBS, and CBS groups, the clinical symptoms at the first registration were least severe in the RS group. This observation is consistent with previous reports showing that CBD had worse prognosis than PSP [18]. The PSPRS score is significantly correlated with the initial symptoms of personality change and asymmetric onset, which are usually more frequent in CBS. The presence of these two symptoms at disease onset could indicate clinical severity.

Many patients in the RS group presented with initial symptoms in the mandatory inclusion criteria, such as falls (58.9 %), bradykinesia (50.9 %), gait disturbance (69.6 %), and ocular symptoms (24.1 %). However, the frequencies of these initial symptoms in the RS group were not distinctive when compared to those in the other groups. Speech disturbance, cognitive decline, personality change, apraxia, asymmetric onset, and unilateral onset were significantly less frequent in the RS group. In a previous cross-sectional study[2] that included 42 patients with pathologically confirmed RS, the most frequent initial symptoms were falls (85.7 %), bradykinesia (75.6 %), ocular symptoms (70.0 %), and cognitive decline (50.0 %). Compared to that study [2], the mandatory inclusion symptoms such as falls, bradykinesia, ocular symptoms and cognitive decline were less frequent in the RS group in our study. Instead, tremor, dysphagia, and asymmetric onset were more frequent in our RS group (tremor: 9.8 %, dysphagia: 2.7 %, asymmetric onset: 17.9 %). This discrepancy could be caused by that RS patients in this paper were clinically diagnosed not pathologically diagnosed. There are no other reports about cross-sectional data of clinical symptoms of pathologically diagnosed PSP. There are no other reports about cross-sectional data of clinical symptoms of pathologically diagnosed PSP. As for longitudinal analysis, the number of cases was small [16].

These variances mainly resulted from disparities in the clinical and pathological diagnoses of PSP. In addition, race-related differences or differences in the medical systems between Western countries and Japan may have affected these results. Notably, the frequency of speech disturbance in the previous study (32.5 %) was similar to that in the current study. However, there was no data regarding personality change and apraxia in the previous study [2]. Further analysis of clinical symptoms is required to clarify the core clinical symptoms of PSP. Hence, based on our data, more attention should be paid to cognitive dysfunction at the disease onset in PSP. In this paper, we focus on the cross-sectional analysis at the time of initial registration. There are still only a few patients that have been followed up, and we believe that longitudinal analysis will be necessary in the future.

### 4.2 PSPRS score progression

The annual progression of PSPRS score from onset to the first registration and the progression rate of PSPRS score between the first and second registrations did not differ among the three groups. However, the PSPRS score was the lowest in the RS group up to the second registration. PSPRS scores in the RS/CBS group were similar to those in the CBS group. This similarity could be because the presence of personality changes worsened the PSPRS score at onset in the CBS and RS/CBS groups. The annual rate of PSPRS score progression from onset to the first registration in the RS group (11.2 ± 8.3) was consistent with that reported previously (9.1 to 11.1) [14], [19], [20].

To our best knowledge, no study has reported the annual PSPRS score progression in CBS. Unlike the annual progression rate of the PSPRS score, the annual reduction rate of the BI score from onset to first registration was the highest in the CBS group. The PSPRS was developed for patients with PSP and included many items corresponding to its various symptoms. Thus, the BI may be more sensitive than the PSPRS for detecting CBS progression. The better clinical course scores in the RS group than that in the CBS group were consistent with previous reports [18]. Nevertheless, a longer follow-up period will be required before conclusions can be drawn based on these results.

Among the symptoms at the first registration, motor dysfunction in sitting down and upward ocular motor movements, as well as cognitive dysfunction (MMSE scores) and frontal lobe dysfunction (grasping/imitation/utilizing behavior, FAB scores), were correlated with PSPRS score progression between the first and second registrations. In previous studies [15], [16], [17], [21], [22], the predictive factors for poor survival in patients with PSP were male sex, older age at onset, sleep disorders, hallucinations, dysphagia, early falls, and dementia. The predictive factors for rapid PSPRS score progression were cognitive impairment, depression, dysphagia for solids or liquids, gait, and returning to a seat [19], [23]. Many non-motor symptoms were found to be predictive factors for disease progression, and consistent results were obtained in the current study. These findings indicate that cognitive function, especially frontal lobe dysfunction, plays a crucial role in predicting the progression of clinical symptoms in PSP.

At the third registration, the PSPRS score did not differ among the three groups and appeared to improve in the RS/CBS group. This observation could be because of the high drop-out rate in patients with severe RS/CBS as a result of hospital transfers or patient deaths. Future analyses with more patients are necessary to determine the long-term course of clinical features.

### 4.3 Mortality after the first registration

The Kaplan–Meier curve analysis (Fig. 2b) showed that the survival rate after onset tended to be lower in the CBS group than in the RS group, similar to previous reports [24], [25]. This study’s 5-year survival rate from disease onset was approximately 90 %. Previously reported 5-year survival rates in Western countries were 70.8 % for RS [24] and 80 % for CBD [25]; in Japan, it was approximately 65 % in 2005 [15]. The median survival time of CBS was 10.0 years, which was longer than that reported (7.0 years) in a pathological analysis of 32 Japanese patients with CBD between 1996 and 2018 [26]. The high 5-year survival rate in the current study could be because of recent improvements in the Japanese medical system, such as the long-term care insurance system or the intractable disease policy introduced by the Japanese government. However, the number of patients followed up for the long term remains small, and further studies are required.

Based on our small number of autopsy patients (n = 21, Table S5), the NINDS-SPSP diagnostic criteria demonstrated low sensitivity (36.4 %) but high specificity (90.0 %), even when potential PSP patients were included. The low sensitivity and high specificity of the NINDS-SPSP diagnostic criteria were consistent with those reported in a previous study that included 66 patients with pathologically confirmed PSP (sensitivity/specificity, 31.1 %/86.8 %) [27]. In contrast, the Armstrong CBD diagnostic criteria (for possible CBS) had a relatively higher sensitivity (66.7 %) and lower specificity (22.2 %) than the NINDS-SPSP diagnostic criteria. Analysis of CBS patients showed a wide variety of underlying pathologies, with approximately half of the patients being CBD and approximately 20 % being PSP[28]. This is consistent with the fact that there are many patients in which the clinical diagnoses of RS and CBS overlap. Most of the CBS patients registered in the JALPAC study were possible CBS and may not actually be CBD. This may be the reason for the low specificity of the Armstrong CBD diagnostic criteria.

However, our data showed lower sensitivity and higher specificity of the Armstrong criteria compared to a previous study that included 32 patients with CBS [29], in which the sensitivity and specificity were 94.7 % and 0.0 %, respectively. In our study, six patients clinically diagnosed with CBS were pathologically diagnosed with PSP, and one patient was pathologically diagnosed with CBD. The six patients with PSP met the mandatory NINDS-SPSP inclusion diagnostic criteria. However, many of them also presented with mandatory exclusion criteria for RS, such as alien limb syndrome, cortical sensory deficits, focal frontal/temporoparietal atrophy, and asymmetric parkinsonian signs. These items might not be suitable as exclusion criteria for improving the sensitivity of PSP diagnosis, although further studies are required to clarify this aspect.

In contrast, only one patient with hallucinations or delusions unrelated to dopaminergic therapy and cortical dementia of Alzheimer’s type was pathologically diagnosed with PSP. The RS or CBS exclusion criteria could be more important than the four items mentioned above. Clinical CBS includes many other diseases associated with dementia in pathological studies. Further analysis of pathologically confirmed patients will be required to clarify the core clinical features of PSP/CBS, and we will continue to confirm the pathological diagnoses of our included patients.

### 4.4 Relationship between PSPRS and BI scores

The PSPRS was developed to evaluate the severity of the neurological symptoms of PSP; it is widely used as an evaluation scale and includes the following six areas: history, mentation, bulbar, ocular motor, limb motor, and gait and midline [14]. The BI is an evaluation tool used to assess activities of daily living and is widely used for patients with stroke and neurodegenerative disorders [30]. It is especially useful as a simple index for scoring improvement during rehabilitation. To our best knowledge, this study is the first to report a strong correlation between PSPRS and BI scores in patients with PSP. Our results indicate that the BI could be an alternative instrument for the long-term evaluation of PSP.

### 4.5 Limitations of the study

First, the number of patients diagnosed based on pathological findings was quite low. Second, in our patients, all CBS patients possible CBS of Armstrong criteria. We aim to register as many patients with atypical parkinsonian syndrome as possible, owing to which there might be a risk of overdiagnosis. Third, there are many overlapping symptoms (falls and hypokinesia) among the three groups of patients, even though hallucinations, apraxia, and laterality were common in the CBS group. It could mean that the criteria for possible PSP/CBS could not fully separate PSP and CBS. Therefore, we must continue longitudinal follow-ups and focus on analyses based on pathological diagnoses. Fourth, we did not use the new PSP criteria [31] that were published after initiating the JALPAC. We will include information corresponding to the new diagnostic criteria in future studies.

## 5 Conclusion

Several patients meet the clinical criteria for both possible RS and CBS. The initial clinical characteristics of RS are generally better than those of other conditions. Cognitive symptoms at the time of the first registration could be another predictive factor for poor prognosis at the second registration. We continue to register patients for the JALPAC to determine the longitudinal clinical courses of RS and CBS and to establish better diagnostic biomarkers based on pathological diagnoses.

## Author contributions

Study conception and design: HT, RH, KN, and TI. Data acquisition, analysis, and interpretation: HT, RH, IA, TS, TT, MM, OO, SM, KH, AT, HK, MK and TN. Statistical analysis: HT and RH. Drafting of the manuscript: HT and RH. Manuscript revision: all authors. All authors approved the final version of the article.

## Funding

This work was supported by the Research Committee on CNS Degenerative Diseases, Research on Policy Planning and Evaluation for Rare and Intractable Diseases, Ministry of Health, Labour and Welfare, Japan [grant number 20FC1049] and AMED [grant number JP22ek0109545]. The funding sources were not involved in study design; in the collection, analysis and interpretation of data; in the writing of the report; and in the decision to submit the article for publication.

## CRediT authorship contribution statement

**Hiroshi Takigawa:** Writing – review & editing, Writing – original draft, Project administration, Methodology, Investigation, Formal analysis, Data curation. **Ritsuko Hanajima:** Writing – review & editing, Writing – original draft, Project administration, Methodology, Investigation, Formal analysis, Data curation, Conceptualization. **Ikuko Aiba:** Writing – review & editing, Investigation, Formal analysis, Data curation. **Takayoshi Shimohata:** Writing – review & editing, Investigation, Formal analysis, Data curation. **Takahiko Tokuda:** Writing – review & editing, Investigation, Formal analysis, Data curation. **Mitsuya Morita:** Writing – review & editing, Investigation, Formal analysis, Data curation. **Osamu Onodera:** Writing – review & editing, Investigation, Formal analysis, Data curation. **Shigeo Murayama:** Writing – review & editing, Investigation, Formal analysis, Data curation. **Kazuko Hasegawa:** Writing – review & editing, Investigation, Formal analysis, Data curation. **Aya M. Tokumaru:** Writing – review & editing, Investigation, Formal analysis, Data curation. **Hisanori Kowa:** Writing – review & editing, Investigation, Formal analysis, Data curation. **Masato Kanazawa:** Writing – review & editing, Investigation, Formal analysis, Data curation. **Tameto Naoi:** Writing – review & editing, Investigation, Formal analysis, Data curation. **Kenji Nakashima:** Writing – review & editing, Project administration, Methodology, Investigation, Funding acquisition, Formal analysis, Data curation, Conceptualization. **Takeshi Ikeuchi:** Writing – review & editing, Project administration, Methodology, Investigation, Funding acquisition, Formal analysis, Data curation, Conceptualization.

## Declaration of competing interest

The authors declare that they have no known competing financial interests or personal relationships that could have appeared to influence the work reported in this paper.
