# Supplementary material for: Japanese longitudinal biomarker study in progressive supranuclear palsy and corticobasal degeneration: Clinical features of the first registered patients and short-term follow-up analysis
Source: Clin Park Relat Disord. 2024 Oct 26;11:100279. doi: 10.1016/j.prdoa.2024.100279 (PMC11554922; doi:10.1016/j.prdoa.2024.100279)
Supplement: Supplementary Data 1 [file mmc1.docx]

**Japanese Longitudinal Biomarker Study in Progressive Supranuclear Palsy and Corticobasal Degeneration: Clinical Features of the First Registered Patients and Short-Term Follow-up Analysis**

**SUPPLEMENTARY MATERIAL**

Standardized protocols for biofluid collection and MR imaging

Blood sample (serum, plasma, genomic DNA and lymphoblastoid cell lines)

Peripheral venous blood was drawn under fasting conditions for collecting serum, plasma, genomic DNA, and lymphoblastoid cell lines. Blood without anticoagulant was centrifuged at 2,000 g for 5min at 4°C, and the serum was collected from the supernatant. Genomic DNA was isolated from the resultant buffy coats using standard procedures. Blood containing EDTA-2Na was centrifuged at 2,000*g* for 5 min at 4°C, and the plasma was collected from the supernatant. Frozen serum and plasma were shipped from each of clinical site to the Biomarker core at Niigata University, and subsequently aliquoted into 0.5 mL polypropylene tubes and kept frozen at −80°C. We have generated lymphoblastoid cell lines using Epstein-Barr virus. The cell lines were maintained in RPMI1640 supplemented with 10% fetal bovine serum.

Cerebrospinal fluid (CSF)

Lumbar puncture was performed in the morning to limit potential circadian fluctuation of CSF proteins and metabolites. The first 2 mL of CSF were sent to a local laboratory for determination of protein, glucose and cell count. Up to 10 ml CSF was then taken from each subject into polypropylene tubes followed by freezing and shipped to the Biomarker core at Niigata University. CSF was aliquoted at a volume of 0.5 mL and stored at −80°C until the assay.

Magnetic resonance imaging (MRI)

Structural MRI included a three-dimensional (3D) sagittal magnetization prepared-rapid gradient echo or inversion-recovery spoiled gradient echo T1-weighted (T1W) sequences acquired with a voxel size of 1.1×1.1×1.2 mm3 as per the ADNI protocol. The full details are described in the ADNI online manual (<http://adni.loni.usc.edu/methods/documents/mri-protocols/>)

Table S1. The JALPAC study group as of December 2020

| **Institution** | **Investigators** |
| --- | --- |
| Tottori University | R Hanajima, H Takigawa |
| Kobe University | T Toda |
| Nagoya University | G Sobue, M Katsuno, N Atsuta |
| Tokyo Metropolitan Neurological Hospital | K Sugaya |
| Jichi Medical University | M Morita, T Naoi |
| Kochi University | Y Osaki |
| National Hospital Organization (NHO) Matsue Medical Center | K Nakashima, Y Adachi, H Kowa |
| NHO Higashinagoya National Hospital | I Aiba |
| Kurashiki Heisei Hospital | Y Takao |
| Misasa Onsen Hospital | Y Morio |
| Niigata University | T Ikeuchi, O Onodera, M Kanazawa |
| Gunma University | Y Ikeda, M Ikeda |
| Kyoto Prefectural University of Medicine | T Tokuda |
| Tokyo Metropolitan Geriatric Institute for Geriatrics and Gerontology | S Murayama, AM Tokumaru, R Sengoku |
| NHO Sagamihara National Hospital | K Hasegawa |
| NHO Chiba-East-Hospital | K Arai |
| Okayama Kyokuto Hospital | K Kashihara |
| Yamanashi University | Y Takiyama, F Kobayashi |
| National Center Hospital, National Center of Neurology and Psychiatry | M Murata |
| Juntendo University | N Hattori |
| Aichi Medical University | M Yoshida |
| Tokushima University | Y Izumi |
| Vihara Hananosato Hospital | M Oda |
| The University of Tokyo | S Tsuji, J Mitsui |
| Mie University | Y Kokubo |
| Chiba University | S Kuwabara, S Hirano |
| Juntendo University Koshigaya Hospital | A Yoritaka |
| Tohoku University | M Aoki, T Hasegawa |
| Okayama University | K Abe, T Yamashita |
| Fukuoka University | Y Tsuboi |
| Osaka University | H Mochizuki |
| Hokkaido University | H Sasaki, I Yabe |
| University of Occupational and Environmental Health, Japan | H Adachi |
| Tokyo Medical and Dental University | T Yokota |
| NHO Hyogo-Chuo Hospital | I Funakawa |
| Toho University Omori Medical Center | O Kano |
| NHO Mie Hospital | A Niwa |
| Fujimi-Kogen Hospital | T Yoshida |
| Yamagata University | N Saito |
| NHO Niigata National Hospital | K Oota |
| Gifu University | T Shimohata |
| NHO Suzuka National Hospital | S Kuru |
| Kuwana City Medical Center | R Sasaki |
| Toho University Sakura Hospital | R Sakakibara |
| Fukushimura Hospital | D Kaneta |

Table S2. Clinical criteria for the diagnosis of PSP/CBS

| RS [10] |
| --- |
| Mandatory inclusion criteria   1. Gradually progressive disorder 2. Onset at age 40 years or later 3. Vertical supranuclear palsy 4. Slowing of vertical saccades 5. Prominent postural instability with falls in the first year of disease onset   Diagnostic certainty  Probable RS: A + B + C + E  Possible RS: A + B + C or A + B + D + E  Mandatory exclusion criteria  Recent history of encephalitis  Alien limb syndrome, cortical sensory deficits, focal frontal, or temporoparietal atrophy  Hallucinations or delusions unrelated to dopaminergic therapy  Cortical dementia of Alzheimer’s type  Prominent, early cerebellar symptoms or prominent, early unexplained dysautonomia  Severe, asymmetric parkinsonian signs  Neuroradiological evidence of relevant structural abnormality  Whipple’s disease, confirmed by polymerase chain reaction if indicated  Supportive criteria  Symmetric akinesia or rigidity, proximal more than distal  Abnormal neck posture, especially retrocollis  Poor or absent response of parkinsonism to levodopa therapy  Early dysphagia and dysarthria  Early onset of cognitive impairment, including at least two of the following: apathy, impairment in abstract thought, decreased verbal fluency, utilization or imitation behavior, or frontal release signs |
| PSP-P [2] |
| Mandatory inclusion criteria   1. Asymmetric onset 2. Tremor 3. A moderate initial therapeutic response to levodopa within the first 2 years   Diagnostic certainty  PSP-P: A + B + C  Mandatory exclusion criteria  Falls, cognitive decline, supranuclear gaze palsy within the first 2 years |
| PSP-PAGF [3] |
| Mandatory inclusion criteria   1. Gradual onset 2. Early freezing of gait or speech   Diagnostic certainty  PSP-PAGF: A + B  Mandatory exclusion criteria  Sustained response to levodopa  Tremor  Imaging changes suggestive of lacunar infarcts or subcortical white matter ischemia suggestive of  Binswanger’s disease  Mandatory exclusion criteria within the first 5 years  Limb rigidity  Dementia  Supranuclear ophthalmoplegia  History of acute focal neurological events due to stroke |
| PSP-C [11] |
| Mandatory inclusion criteria   1. Gradually progressive disorder 2. Onset at age 40 years or later 3. Vertical supranuclear palsy 4. Truncal and limb cerebellar ataxia within the first 2 years 5. Postural instability with falls within the first 2 years   Diagnostic certainty  Probable PSP-C: A + B + C + D + E  Possible PSP-C: A + B + D + E  Mandatory exclusion criteria  Urinary incontinence (inability to control the release of urine from the bladder, with erectile dysfunction in males)  An orthostatic decrease of blood pressure within 3 min of standing by at least 30 mmHg systolic or 15 mmHg diastolic  “Hot-cross bun” sign on head magnetic resonance imaging |
| Modified Cambridge CBD criteria [12] |
| Mandatory inclusion criteria   1. Insidious onset and gradual progression 2. No sustained response to levodopa treatment   Major and minor criteria  Motor features   1. Akinetic rigid syndrome 2. Focal or segmental myoclonus 3. Asymmetrical dystonia   Cortical motor sensory features   1. Limb apraxia 2. Alien limb phenomenon 3. Cortical sensory loss or dyscalculia   Cognitive features   1. Speech and language impairments 2. Frontal executive dysfunction 3. Visuospatial deficits   Diagnostic certainty  CBS: A + B + two major criteria (C, F, I) + two minor criteria (D, E, G, H, J, K) |
| Armstrong’s CBD criteria [13] |
| Mandatory inclusion criteria   1. Insidious onset and gradual progression 2. Minimum duration of symptoms: 1 Year 3. Age at onset ≥ 50 years 4. No family history   Mandatory exclusion criteria  Evidence of Lewy body disease: classic 4-Hz Parkinson’s disease resting tremor, excellent and sustained levodopa response, or hallucinations  Evidence of multiple system atrophy: dysautonomia or prominent cerebellar signs  Evidence of amyotrophic lateral sclerosis: presence of both upper and lower motor neuron signs  Semantic- or logopenic-variant primary progressive aphasia  Structural lesion suggestive of focal cause  Granulin mutation or reduced plasma progranulin levels, TDP-43 mutations, FUS mutations  Evidence of Alzheimer’s disease: strongly suggestive laboratory findings, such as low CSF Aβ42/tau ratio or positive 11C–Pittsburgh compound B PET  Genetic mutation suggesting Alzheimer’s disease (e.g., presenilin, amyloid precursor protein mutations)  Diagnostic certainty  Clinical research criteria for probable sporadic CBD: A + B + C + D + exclusion genetic mutation affecting (e.g., MAPT mutation)  Clinical subtype  1) Probable CBS or 2) frontal behavioral-spatial syndrome (FBS) or non-fluent/agrammatic variant of primary progressive aphasia (NAV) or 3) PSPS + ≥ one CBS criteria (E, F, G, H, I, J)  Clinical research criteria for possible CBD: A + B  Clinical subtype  1) Probable CBS or 2) FBS or NAV or 3) PSPS + ≥ one CBS criteria (F, G, H, I, J)  Clinical subtype   1. CBS 2. Limb rigidity or akinesia 3. Limb dystonia, 4. Limb myoclonus 5. Orobuccal or limb apraxia 6. Cortical sensory deficit 7. Alien limb phenomena (more than simple levitation)   Diagnostic certainty  Probable CBS: Asymmetric presentation of any two criteria (E, F, G) + any two criteria (H, I, J)  Possible CBS: presentation of any one criterion (E, F, G) + one criterion (H, I, J)   1. FBS 2. Executive dysfunction 3. Behavioral or personality changes 4. Visuospatial deficits   Diagnostic certainty  FBS: any two criteria (K, L, M)   1. NAV 2. Effortful, agrammatic speech 3. Impaired grammar/sentence comprehension with relatively preserved single word comprehension 4. Groping, distorted speech (apraxia of speech)   Diagnostic certainty  NAV: N + one criteria (O, P)   1. Progressive supranuclear palsy syndrome (PSPS) 2. Axial or symmetric limb rigidity or akinesia 3. Postural instability or falls 4. Urinary incontinence 5. Behavioral changes 6. Supranuclear vertical gaze palsy or decreased velocity of vertical saccades   Diagnostic certainty  PSPS: three criteria (Q, R, S, T, U) |

Table S3. Clinical datasheet for 1^st^ registration

| **Item** | **Description** |
| --- | --- |
| Age of registration | Age, in years, at the time of registration in the study |
| Sex | Male/female |
| Medical history | History of encephalitis, brain injury, or exposure to [1-methyl-4-phenyl-1,2,3,6-tetrahydropyridine](https://ejje.weblio.jp/content/1-methyl-4-phenyl-1%2C2%2C3%2C6-tetrahydropyridine) (MPTP) |
| Family history | History of similar disease in a blood relative, consanguineous marriage |
| Age of onset | Age, in years, at the time of the first reported symptom considered to be attributable to PSP/CBD |
| Clinical course | Progressive clinical course, continuous asymmetric symptoms (for less than 3 years), continuous asymmetric symptoms (for over 3 years), history of acute focal neurological events due to stroke in the first 5 years, and history of repeated strokes with the step-wise progression of parkinsonian features |
| Initial symptoms | Presence of the following clinical symptoms at the time of onset: falls, bradykinesia, gait disturbance, tremor, ocular symptoms, speech disturbance, dysphagia, cognitive decline, hallucination, abnormal behavior, personality change, depression, apraxia, traffic accident, urinary disturbance, dizziness, asymmetric onset, or unilateral onset |
| Clinical symptoms at the 1^st^ registration | External ocular movement disturbance, postural instability, dementia, hallucination/delusion, extrapyramidal sign, cerebellar sign, autonomic disturbance, cortical symptoms, behavioral change, motor neuron signs |
| Neuroimaging | Structural magnetic resonance imaging (MRI) findings, including neuroradiologic evidence of relevant structural abnormalities (i.e., basal ganglia or brainstem infarcts, lobar atrophy) |
| Laboratory data | Whipple’s disease confirmed using polymerase chain reaction; mutations in the microtubule associated protein tau (MAPT) gene, granulin (GRN) gene, TAR DNA-binding protein 43 (TDP-43) gene, fused in sarcoma (FUS) gene, presenilin gene, or amyloid precursor protein gene; decrease of the level of amyloid beta and tau in cerebrospinal fluid |
| Response of therapy | Response to levodopa was graded using a 4-point scale as follows: 1 = nil, or slight response (< 30% improvement), 2 = moderate response (30–50% improvement), 3 = good response (51–70% improvement), 4 = excellent response (71–100% improvement), the period of illness judged the response of levodopa, continuousness of the response to levodopa, presence of sever chorea movements due to levodopa, and taking or not of neuroleptic agent at onset |
| Others | Presence of optokinetic nystagmus, and the grade of manual muscle testing in deltoid muscle, extensor digitorum muscle and iliopsoas muscle |

Table S4. Changes in PSPRS scores after the first registration

|  | Registration | Second | Third | Fourth |
| --- | --- | --- | --- | --- |
| Group |  |  |  |  |
| RS | Re-registration | 32 | 25 | 17 |
|  | Death | 3 | 0 | 4 |
|  | Transfer to another hospital | 12 | 6 | 1 |
|  | Duration since first registration, years | 1.3 ± 0.4 | 2.2 ± 0.4 | 3.3 ± 0.4 |
| RS/CBS | Re-registration | 19 | 11 | 5 |
|  | Death | 4 | 1 | 1 |
|  | Transfer to another hospital | 9 | 4 | 1 |
|  | Duration since first registration, years | 1.3 ± 0.7 | 2.4 ± 0.5 | 3.5 ± 0.4 |
| CBS | Re-registration | 65 | 32 | 15 |
|  | Death | 13 | 8 | 5 |
|  | Transfer to another hospital | 22 | 6 | 6 |
|  | Duration since first registration, years | 1.4 ± 1.0 | 2.2 ± 0.4 | 3.1 ± 0.2 |

PSPRS: Progressive Supranuclear Palsy Rating Scale; RS: clinical Richardson’s syndrome; RS/CBS: Richardson’s syndrome/corticobasal syndrome; CBS: corticobasal syndrome

Table S5. Clinical and pathological diagnoses in autopsy cases

|  |  | Pathological diagnosis  Clinical diagnosis | PSP | CBD | Other | Number | Number |
| --- | --- | --- | --- | --- | --- | --- | --- |
| PSP  (RS) |  | RS | 2 | 0 | 0 | 5 | 2 |
|  | CBS | RS/CBS | 2 | 0 | 1^a^ |  | 16 |
|  |  | CBS | 6 | 1 | 5^b^ | 16 |  |
|  |  | PSP-P and CBS overlap | 0 | 1 | 0 |  |  |
|  |  | Not applicable for RS/CBS | 1 | 1 | 1^c^ |  | 3 |
|  |  | sum | 11 | 3 | 7 | 21 | 21 |

a: Alzheimer’s disease

b: Alzheimer’s disease, Lewy body disease, frontotemporal lobar degeneration with TAR DNA-binding protein of 43 kDa proteinopathy (FLDP-TDP), FLDP-TDP type B + Lewy body dementia, and Kii-parkinsonism-dementia complex

c: Frontotemporal lobar degeneration

PSP: progressive supranuclear palsy; RS: Richardson’s syndrome; RS/CBS: Richardson’s syndrome/corticobasal syndrome

CBS: corticobasal syndrome

**Supplementary Figure**


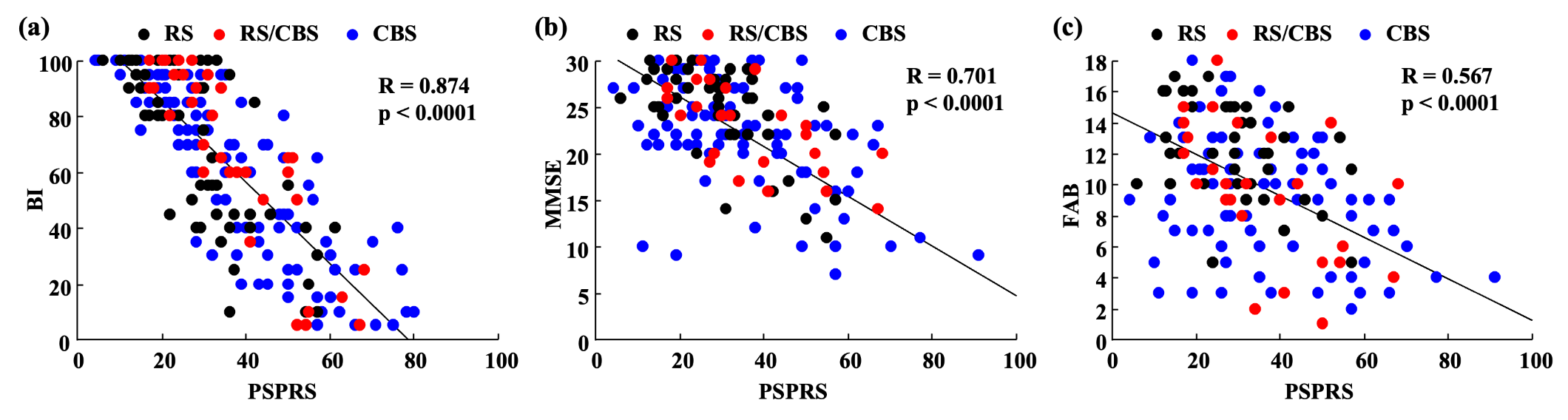


Fig. S1 Correlations of PSPRS score with BI, MMSE, and FAB scores

Pearson’s correlation coefficient is used to assess the correlations between the scores of all patients. a) The PSPRS and BI scores are very strongly correlated (p < 0.0001, R = 0.874). b) The PSPRS and MMSE scores are strongly correlated (p < 0.0001, R = 0.701). c) The PSPRS and FAB scores are correlated (p < 0.0001, R = 0.567).

PSPRS: Progressive Supranuclear Palsy Rating Scale, BI: Barthel Index, MMSE: Mini-Mental State Examination, FAB: Frontal Assessment Battery, RS: Richardson’s syndrome, RS/CBS: Richardson’s syndrome/corticobasal syndrome, CBS: corticobasal syndrome
